# Extended two-photon microscopy in live samples with Bessel beams: steadier focus, faster volume scans, and simpler stereoscopic imaging

**DOI:** 10.3389/fncel.2014.00139

**Published:** 2014-05-20

**Authors:** Gabrielle Thériault, Martin Cottet, Annie Castonguay, Nathalie McCarthy, Yves De Koninck

**Affiliations:** ^1^Département de Physique, de Génie Physique et d'Optique, Centre d'Optique, Photonique et Laser, Université LavalQuébec, QC, Canada; ^2^Centre de Recherche de l'Institut Universitaire en Santé Mentale de QuébecQuébec, QC, Canada; ^3^Département de Psychiatrie et de Neurosciences, Université LavalQuébec, QC, Canada

**Keywords:** nonlinear microscopy, depth of field, axicon, nondiffractive beam, temporal resolution, 3D imaging, functional calcium imaging, cellular imaging

## Abstract

Two-photon microscopy has revolutionized functional cellular imaging in tissue, but although the highly confined depth of field (DOF) of standard set-ups yields great optical sectioning, it also limits imaging speed in volume samples and ease of use. For this reason, we recently presented a simple and retrofittable modification to the two-photon laser-scanning microscope which extends the DOF through the use of an axicon (conical lens). Here we demonstrate three significant benefits of this technique using biological samples commonly employed in the field of neuroscience. First, we use a sample of neurons grown in culture and move it along the z-axis, showing that a more stable focus is achieved without compromise on transverse resolution. Second, we monitor 3D population dynamics in an acute slice of live mouse cortex, demonstrating that faster volumetric scans can be conducted. Third, we acquire a stereoscopic image of neurons and their dendrites in a fixed sample of mouse cortex, using only two scans instead of the complete stack and calculations required by standard systems. Taken together, these advantages, combined with the ease of integration into pre-existing systems, make the extended depth-of-field imaging based on Bessel beams a strong asset for the field of microscopy and life sciences in general.

## Introduction

Since its invention in 1990, two-photon microscopy (Denk et al., 1990) has become an essential tool for biologists, especially in the field of neuroscience (Zipfel et al., 2003). It can reveal structures deep inside tissue (Helmchen and Denk, 2005), and fluorescent markers can help track activity in networks of cells (Stosiek et al., 2003; Lütcke and Helmchen, 2011). The intrinsic optical sectioning of two-photon microscopy limits the focal volume to a very thin plane, which has been exploited to improve axial resolution and limit photo damage around the focal volume (Zipfel et al., 2003). When the features of interest are mainly located in the same plane or when a volume sample is densely labeled, optical sectioning is a great advantage. But if the labeling is sparse and the cells are distributed at different depths in an extended volume, optical sectioning forces the use of integrating multiple frames at different depths to recover all the information. This limits the temporal resolution of the measurements. Optical sectioning therefore poses challenges for scanning large volumes, in particular for functional cellular imaging in live tissue or reconstructions of large structures. Many research groups are attempting to address this challenge (Göbel et al., 2007; Otsu et al., 2008; Reddy et al., 2008; Grewe et al., 2010; Botcherby et al., 2012). Furthermore, a small depth of field (DOF) can become problematic when the sample moves vertically, as it often occurs during *in vivo* measurements (Laffray et al., 2011).

In two-photon microscopy, it is possible to extend the DOF of the system by generating a nondiffracting beam at the sample, while maintaining a good transverse resolution throughout the sample. Different approaches have been recently proposed to shape the distribution of light at the sample into a Bessel-Gauss beam (Botcherby et al., 2006; Dufour et al., 2006; Thériault et al., 2013), which is characterized by an intense central lobe and is nondiffractive, i.e., the central lobe has a constant radius.

Although highly promising, these previous reports of two-photon microscopy with an extended DOF have only shown results with powerful fluorescent samples, such as fluorescent micro-beads or stained pollen grains. In order to demonstrate to the neuroscience community that the Bessel extended DOF microscope is suitable to this field, biologically relevant samples must be used. To our knowledge, this paper is the first report of such measurements.

In this paper, we demonstrate experimentally three advantages of two-photon microscopy with an extended DOF using a Bessel beam when compared to standard two-photon microscopy. These benefits are: (1) a more robust focus when sample moves in the z direction, (2) an increase in information throughput or in scanning speed for volume samples, and (3) the possibility of creating stereoscopic images with only two x-y scans, dramatically reducing the number of scans required to examine the relationship between structures in the axial direction. We illustrate each of these advantages with a set of measurements performed on different biological samples that are commonly used in neuroscience.

## Materials and methods

### Configuration of a bessel EDOF two-photon microscope

**Figure 1:**
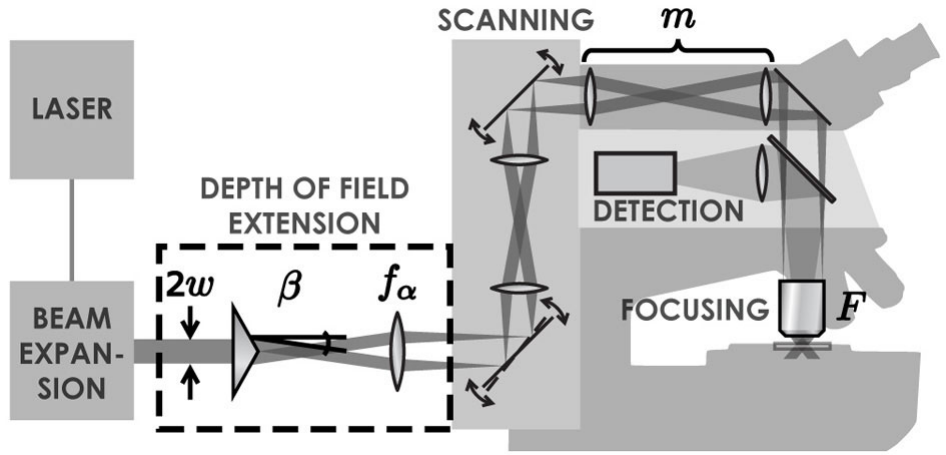
**Illustration of the set-up**. A Ti:Sapphire laser generates an ultra-short pulsed laser beam with a Gaussian profile. This beam is expanded with a simple two-lens telescope. Once expanded, the beam passes through an axicon followed by a lens. These two elements transform the laser beam into an annulus of light. This annulus is imaged into the back focal plane of the objective lens, which creates a tightly focused Bessel-Gauss beam in the sample. The scanning system enables a beam tilt in the back focal plane of the objective, leading to an x-y scan of the beam in the sample. Fluorescence light is retro-collected with the objective and directed to a photomultiplier tube with a dichroic mirror.

Standard two-photon microscopes can easily be modified to extend the DOF with a Bessel beam by placing an axicon and a lens in the laser beam path (Figure 1). Axicons are refractive optical elements shaped as a cone (McLeod, 1954), which deviate light toward the optical axis by an angle β simply calculated from Snell's refraction law. The complete details of this method are presented in a previous paper (Thériault et al., 2013).

Let us quickly recall the parameters of the focal line in the extended DOF system. The transverse resolution ρ and the DOF *L* of the two-photon excitation at full-width at half maximum are given by:

(1) $$\rho =m\frac{1.629\lambda }{2\pi \sin\beta }\text{ and }L=m^{2}\frac{0.577w}{\tan\beta }$$

where λ is the wavelength of the laser beam and *m* = *Ff*_1_/*f*_2_*f*_α_ is the magnification applied to the Bessel beam while being relayed to the sample, with focal lengths *F* of the microscope objective, *f*_1_ and *f*_2_ of relay lenses in the scanning system and *f*_α_ of the lens after the axicon. The advantage of this approach is that it allows adjusting the DOF independently (without changing the resolution) by using a simple telescope (Thériault et al., 2013).

### Custom-built extended DOF microscope

In this paper, experiments were carried out on two different systems. The first one is a custom-built laser-scanning microscope, which includes a removable DOF extension module as illustrated in Figure 1.

**Figure 2:**
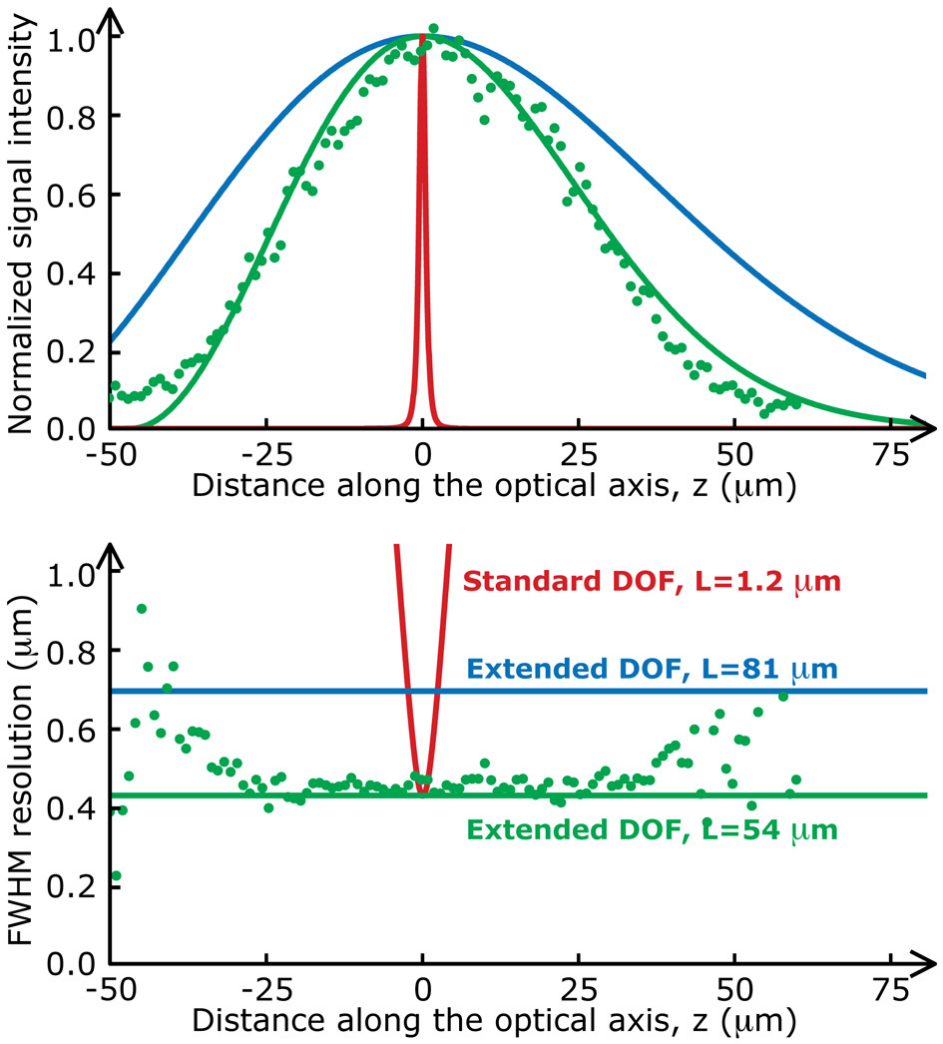
**Two-photon fluorescence distributions for the data presented in this paper**. The on-axis intensity (**top**) and the transverse resolution (**bottom**) of a standard two-photon set-up (red lines) varies much more rapidly along the optical axis than those of the Bessel extended DOF two-photon microscope (green and blue lines). Green dots are experimental values measured with fluorescent microspheres (*Molecular* *Probes*, Fluosphere 505/515, diameter 200 nm) mounted on a coverslip with fluorescent mounting medium (*Dako*).

We used a Ti:Sapphire pulsed laser with central wavelength λ = 900 nm (Chameleon, Coherent), relay lenses with a magnification factor, *f*_1_/*f*_2_ = 1.5 and an objective with a focal length *F* = 4.11 mm (Zeiss, W N-Achroplan 40×, 0.75 *NA*). To demonstrate the flexibility of the Bessel extended DOF set-up, we used different sets of parameters throughout this paper. These parameters are detailed in Table 1. Note that the transverse resolutions achieved with this beam are significantly better than with a Gaussian beam (0.5 μm). The ability to design an optical system with an improved transverse resolution represents an added advantage of this imaging configuration (April et al., 2012). The two-photon signal intensity distributions (on-axis intensity and transverse resolution) for each of these sets of parameters are presented in Figure 2. Experimental measurements with micro-beads show an excellent agreement with the theoretical curves.

**Table 1:** **Components and focal line parameters used for the experiments in this paper that were carried out on the custom-built set-up**.

**Type of DOF**	**Figure**	***w* (mm)**	**Axicon**	***f*_α_ (mm)**	***ρ* (μm)**	***L* (μm)**	**P/*L* (mW/μm)**
Extended	4, 7, 8	1.0	5°, UVFS	45	0.43	54	71/54
	6	2.3	1°, BK7	125	0.69	81	83/81
Standard	4, 6	2.3	N/A	N/A	0.43	1.2	24/1.2

Table 1 also includes the average laser power used for each experiment, measured at the sample plane. Laser power is an important factor in extended DOF imaging because the fluorescence signal intensity is highly dependent on the length, *L* (Thériault et al., 2013). One can also note that for a focal line of approximately 50 μm, roughly 3 times more power was required with the extended DOF set-up to obtain signal-to-noise ratios similar to when using the standard two-photon set-up with the same transverse resolution. Although more power is sent to the sample during one scan, the volume in which this power is focused is much larger than in the conventional set-up. Therefore, the power per unit volume and the peak intensity of the excitation beam are the same as with the standard two-photon microscope to generate the same fluorescence signal. Therefore, photobleaching and photodamage are not an issue.

### Modified commercial microscope

The second system was a Zeiss LSM510 coupled to a Ti:Sapphire pulsed laser (Chameleon, Coherent) for two-photon imaging. We modified this system by adding a simple double-convex lens (*f* = 200 mm, Thorlabs) and an axicon (0.1°, UVFS, Altechna) just before the laser injection porthole. With these parameters and using an objective with a focal length *F* = 4.11 mm (Zeiss, W N-Achroplan 40×, 0.75 *NA*), the system produces a focal line with a transverse resolution of ρ = 1.1 μm and a DOF of *L* = 25 μm. This resolution is not optimal because of the mismatch between the characteristics of the axicon that we had available and the objective lens. An axicon of 0.2° would have yielded a resolution of 0.44 μm. With this DOF, the laser power at the sample for these experiments was 30 mW spread over the 25 μm of the DOF.

This commercial microscopy system also supports multiple modalities such as confocal imaging and a photon-counting unit for fluorescence lifetime imaging (FLIM), which we used for the increased information throughput demonstration. The confocal modality was set at a wavelength of 488 nm and with a pinhole opening of 1 Airy unit (0.61 λ/*NA*).

### Sample preparation

As mentioned in Introduction, we present here three advantages of the Bessel extended DOF microscope, using three different types of samples commonly used in neuroscience. To demonstrate the improvement of focus stability, we use a thin sample, i.e., neurons grown on a glass coverslip and transfected with a fluorescent protein. To demonstrate the increased speed for volumetric scans, we use thick acute slices of adult mouse cortex, stained with a calcium indicator. Finally, to show the simplicity of stereoscopic imaging, we use a fixed sample of mouse cortex, containing fluorescent protein-labeled neurons. In this section, we detail how each type of sample was prepared.

#### Cultured cells

Primary dissociated neurons grown in culture were obtained as described previously (Nault and De Koninck, 2010). Cells were plated at 0–3 post-natal days at a density of approximately 1–2 M cells per coverslip. From day 5, Ara-C (10 μM) was added to the culture medium to kill cells in division and prevent proliferation of glial cells. For DOF stability experiments, mEGFP plasmid was transfected in hippocampal cells in culture at 12 days *in vitro* using lipofectamin 2000 (Invitrogen) and 0.5 μg of DNA per coverslip. Cells were allowed to express the fluorescent protein for 24 h before PFA fixation. Coverslips were fixed in a 4% paraformaldehyde solution with PB 0.1 M at 37°C for 10 min. Fixation was followed by 3 washes (10 min) in PB 0.05 M and coverslips were mounted on glass slides using DAKO fluorescent mounting medium.

#### Live acute brain slices

Acute slices were prepared using the following method. Adult mice were deeply anesthetized with isoflurane and decapitated. The brain was quickly removed and placed in ice-cold solution (≤ 4°C) containing (in mM) 252 sucrose, 2.5 KCl, 1.5 CaCl_2_, 6 MgCl_2_, 10 glucose, 26 NaHCO_3_, 1.25 NaH_2_PO_4_, and 5 kynurenic acid (sACSF). Coronal slices of cortex were cut at 300 μm using a vibratome (VT 1200S, Leica) and kept in artificial cerebro-spinal fluid (ACSF) containing (in mM) 126 NaCl, 2.5 KCl, 2 MgCl_2_, 2 CaCl_2_, 1.25 NaH_2_PO_4_, 26 NaHCO_3_, and 10 glucose, bubbled with 5% CO_2_/95% O_2_ to adjust the pH to 7.4. Slices were incubated with a solution containing 1 μM Fluo-4 AM (Molecular Probes) for 30–60 min at room temperature prior to imaging. To prevent mechanical damage, the slices were placed on a mesh of nylon in a covered 12-well plate, which was continuously bubbled. Slices were then transferred to a recording chamber continuously perfused with ACSF or high potassium solution for recordings to raise network activity. The high K^+^ solution contained: 80 NaCl; 50 KCl; 2 CaCl_2_; 1 MgCl_2_; 25 d-Glucose; 26 NaHCO_3_; 1.25 NaH_2_PO_4_, also gassed with 5% CO_2_/95% O_2_to adjust the pH to 7.4.

#### Fixed tissue preparations

For comparison of images obtained from fixed tissue, 300 μm-thick brain slices from Thy1::COP4-EYFP (Jackson Laboratories) mice and whole dorsal root ganglions (DRGs) from C57 wildtype mice 6 weeks after injection at P6 of a AAV9 viral vector encoding for a GFP (U. Pennsylvania) were fixed by immersion in 4% paraformaldehyde for 1 h.

## Results

### Steadier focus

In standard two-photon microscopy, the DOF is approximately *L* ≈ λ/*NA*^2^ when the back aperture of the objective is properly filled (when the ratio of the objective back aperture to the beam width is π/2). This means that at λ = 900 nm and with a 0.8 *NA* objective, then *L* = 1.4 μm. Such a thin optical section offers a number of advantages, including the ability to resolve small features in three dimensions, but it also comes with drawbacks, such as not being able to resolve complete neurons in a single frame and important fluorescence signal fluctuations due to small perturbations along the vertical axis.

**Figure 3:**
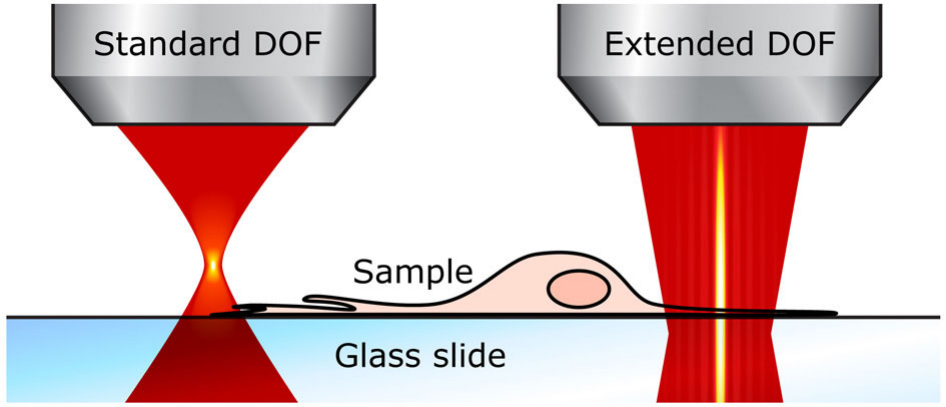
**Extended DOF for imaging thin samples**. With the standard two-photon microscope (left), the focal plane is very thin and small perturbations affect the fluorescence signal. With an extended DOF (right), the focus is much more robust.

Using an extended DOF counters both of these drawbacks. First, the entire cells can be imaged in a single x-y scan. For example, even in thin samples like neurons grown in cultures, fine structures such as dendrites are generally located on the same plane close to the substrate, but they also grow around and above thicker cell bodies, often 10–15 μm thick, which means that the total sample thickness is well over the standard DOF; the different neurites end up not all located in the same plane and thus cannot be captured in one scan (see Figure 3). With an extended DOF of only 15–20 μm, complete cell bodies including the dendrites can be imaged in only one frame. Although this improvement does not imply a very high gain in acquisition speed, it guarantees that all the features of interest are imaged.

Second, small perturbations in the z direction and focus drift do not affect the fluorescence signal when using an extended DOF microscope. Focus drift is a major issue for live-cell imaging (Waters, 2007) and it is often caused by changes in temperature, an unstable stage or focusing mechanism, an uneven perfusion, or movement of the specimen. Post-processing can be used for data with a focus drift in the illumination plane, but when the drift is vertical, the data are lost. This data loss is less an issue in systems that include an autofocus control, which can compensate for slow vertical drifts.

Finally, let us remark that it is also possible to slightly increase a system's DOF by using an objective with a lower numerical aperture (or, equivalently, under-filling the objective back-aperture). For example, as mentioned above, at λ = 900 nm and *NA* = 0.8, the DOF is *L*≈ 1.4μm. But with a numerical aperture of 0.3, the same system has a much larger DOF: *L* ≈ 10μm. However, the major drawback of this approach is that the width of the focal spot also increases: the transverse resolution in this examples goes from 0.56 to 1.5 μm, when calculated with the Abbe criterion: ρ = λ/2 *NA*. With a Bessel beam, in contrast, the transverse resolution is not compromised and remains constant, even when the DOF is extended.

#### Demonstration of focus stability

To demonstrate the enhanced focus stability provided by the extended DOF modification, we used a thin sample of neuron cultures grown on a glass coverslip that we moved in the z direction. Although two-photon microscopy is rarely needed to image cultured samples, two-photon excitation was used here because it offers several advantages: (1) it reduces the sidelobes in the Bessel beam to a point where they are negligible; (2) it maximizes transverse resolution (in fact, the Bessel beam offers even a better resolution than the Gaussian beam; April et al., 2012); (3) using a pulsed laser is ideal for certain imaging modalities, such as fluorescence lifetime imaging (FLIM) (Doyon et al., 2011). In this section, we will compare the acquired images with a standard DOF system to the ones acquired with a Bessel-modified 2P microscope.

**Figure 4:**
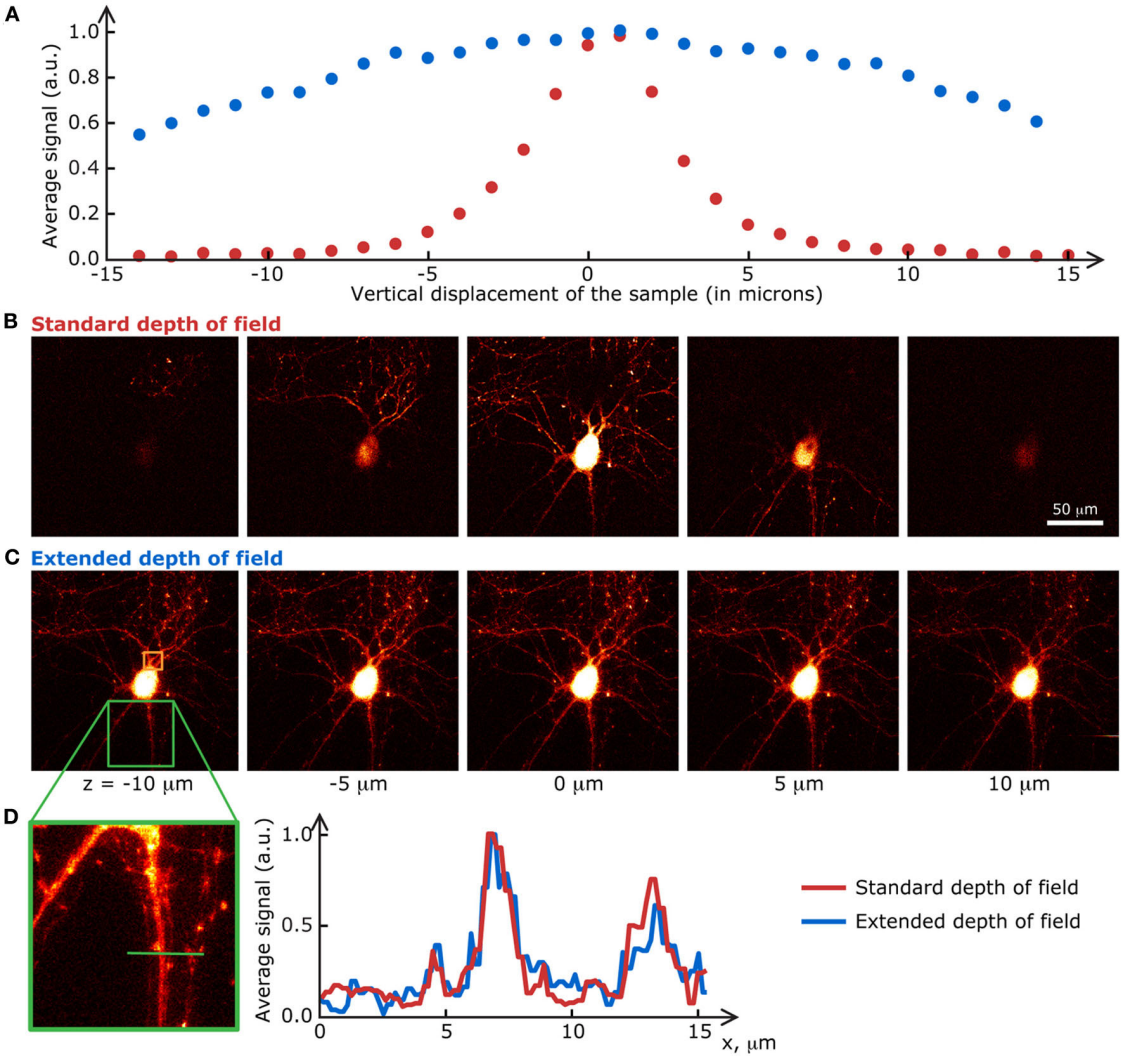
**Extended DOF microscopy provides a steadier focus**. **(A)** Average fluorescence signal from each frame in a z-stack of fixed neurons, grown on a glass coverslip. The stage holder was translated 1 μm in the z direction between each frame. The signal in the extended DOF set-up (blue) is stable over a larger distance than the signal in the standard set-up (red). Single frames at 5 different positions in the z-stack are presented below, for **(B)** the conventional set-up and **(C)** the Bessel extended DOF set-up. **(D)** The DOF extension does not affect the transverse resolution of the system, as shown by this comparison of average intensities from 10 frames for each method.

The biological samples used in this section are neurons grown on a glass coverslip and transfected with a fluorescent protein. Their preparation is detailed in Materials and methods. Fluorescence images of the transfected cells were taken at a speed of 1 s/frame (2 ms/line; 512 × 512 pixels) with the extended DOF set-up described in Materials and methods. The excitation and emission light were separated by a dichroic mirror at 665 nm (Semrock). The emission light was truncated by a 633 nm short-pass filter (Semrock). Between each image, the motorized stage supporting the sample was translated 2 μm in the z direction. The single frames shown in Figure 4 are the raw fluorescence images, only brightness and contrast levels were adjusted. For the graph in Figure 4A, the pixel values on each frame were averaged, and the data was normalized to the maximum of each curve.

At first glance, these results prove that the signal-to-noise ratio of the extended DOF set-up is sufficient to discern single neurites, even though the laser power is spread in a long focal line instead of a tight spot. Furthermore, measurements taken with the extended DOF system can be considered more stable than with the standard two-photon microscope, since external factors such as vibrations of the stage or focus drift do not affect the fluorescence signal intensity, within a range of a few tens of microns, whereas displacements of only a few microns induce dramatic changes in the standard two-photon set-up. This greater signal stability in turn leads to less variability in the measured fluorescence levels, but a possible drawback from this approach could be a higher chance of recording from several dendrites belonging to different cells at the same time if they are superimposed in the z axis.

Finally, *in vivo* measurements could also benefit from the steadier focus that the extended DOF approach provides. For example, when imaging spinal dorsal horn or brain stem features *in vivo* in rats, breathing and cardiac movements induce vertical displacements of tens of microns (Laffray et al., 2011). These displacements make out-of-focus images unusable, which dramatically reduces the temporal resolution. With an extended DOF, all the acquired images can be used if the excitation line is set long enough to keep the features of interest within the excitation volume.

### Faster volume scans

In many biological experiments, the features of interest are spread out in a three-dimensional matrix. It is not always necessary to know at which depths these features are, only their presence/absence, action/reaction or growth/retraction can provide precious information. In such cases, a method to scan the entire volume faster than raster-scanning each plane of interest could be very useful. We demonstrate here that using the Bessel beam extended DOF set-up to image thick live samples leads to a greater throughput of information (more cells sampled) or faster volumetric scans than when using a standard two-photon microscope.

Various approaches can be envisaged when one needs to increase the number of cells sampled within a specific time-frame. Setting the sample's geometry and staining density aside, the approaches can be resumed by two categories: increasing the scanning speed or the excitation volume.

**Figure 5:**
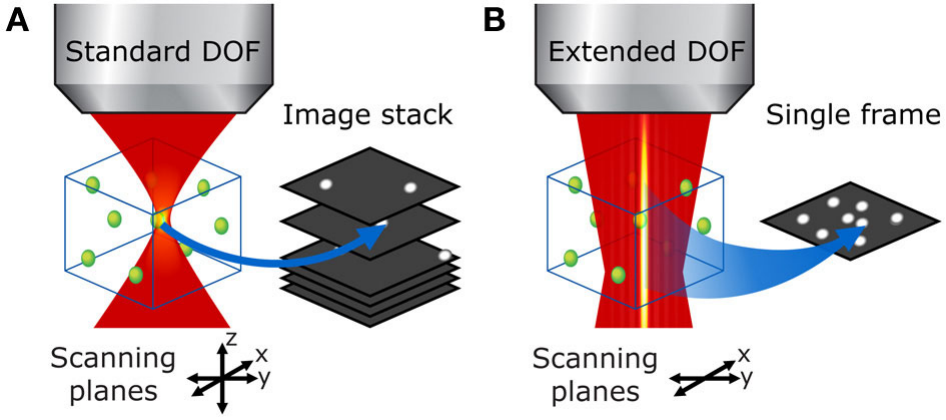
**Bessel beam 3-D raster scan vs. conventional DOF volumetric imaging in a thick sample**. **(A)** With the standard two-photon microscope, the focal volume is very small and one must acquire a stack of raster-scanned images to image the entire volume of interest. **(B)** With an extended DOF, the entire volume of interest can be examined in a single x-y scan, which leads to much faster volumetric scans without compromise on transverse resolution.

Many techniques have been recently developed to increase the scanning speed in two-photon microscopy (Lillis et al., 2008; Otsu et al., 2008; Reddy et al., 2008; Grewe et al., 2010; Truong et al., 2011; Botcherby et al., 2012; Katona et al., 2012). Nevertheless, the most commonly used method to image thick samples still remains the 3-D raster scan (Figure 5). This method consists in rapidly tilting the laser beam with a set of mirrors. Particular mirrors that are extremely fast can also be used to increase scanning speeds [ex.: rotating polygon (Rajadhyaksha et al., 1999), resonant mirrors (Göbel et al., 2007)]. The two set-ups that we used in this paper feature the slower but more adaptable galvanometric mirrors. With these mirrors, line scans of up to 120 Hz can be achieved (for a 4.3 Hz frame-rate with 512 × 512 pixels). Nevertheless, all the results presented here could be reproduced on systems with faster scanning mirrors.

Shaping the laser beam into a Bessel-type nondiffractive beam as we implemented in our two set-ups is a technique that increases the excitation volume. The excitation spot, now spread out into a thin, long line, generates fluorescence signal at various depths simultaneously inside the sample. This way, many cells are sampled in a single x-y scan, even when they are located at different depths.

#### Demonstration: fluorescence lifetime imaging of dorsal root ganglions with 2-photon extended DOF

**Figure 6:**
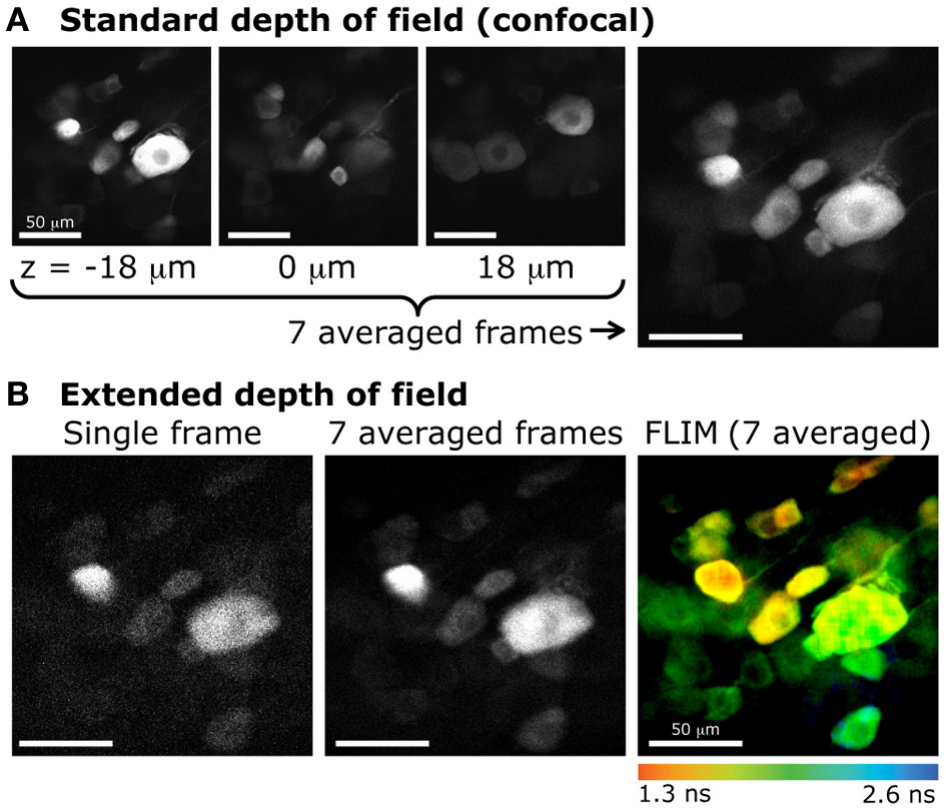
**Extended DOF for fluorescence lifetime imaging of dorsal root ganglion neurons (DRGs)**. **(A)** Confocal imaging of DRG neurons expressing GFP. Images obtained at various focal depths, with a DOF of 2.1 μm. The image on the far right is the resulting z-projection of 7 images taken every 6 μm, between *z* = −18 μm and *z* = 18 μm (example images on the left). **(B)** Extended depth of field with 2-photon excitation and fluorescence lifetime imaging. The same sample was imaged with 2-photon excitation. Left, single frame obtained with a DOF of 20 μm. Center, average of 7 frames (same acquisition time as for each confocal image). Right, color-coded lifetime image of DRG neurons obtained from photons accumulated from 7 consecutive frames.

One application of the extended 2-photon DOF in thick samples would be for the acquisition of fluorescence lifetime data, using the pulsed characteristics of the 2-photon laser for synchronization. In the example provided, lifetime of a fluorescent protein was acquired in DRG neurons (Figure 6).

Furthermore, the extended DOF set-up was implemented on a commercial laser-scanning microscope, by simply adding and aligning the lens and the axicon in the two-photon laser path between the periscope and the microscope. This configuration allowed for measurements of multiple cells located in a large volume. When comparing these measurements to confocal images, where the DOF is very small, we can see that much fewer cells are sampled in a single small-DOF image (Figure 6). As mentioned above, this configuration also allows for fluorescence lifetime imaging. In live tissue, time-lapse fluorescence lifetime imaging could be achieved. Doing so would permit to probe more cells than with the conventional two-photon sectioning and ensure that a maximum of cells would stay in the focal plane throughout the recording.

The extended DOF modification to a two-photon setup also offers a significant increase in speed, especially for imaging cellular dynamics in live tissue. Indeed, a 40 μm thick volume can be completely examined with a single scan. A confocal setup would however require 20 x-y frames with a typical 2 μm-DOF, resulting in a 20-fold increase in acquisition time. This alone allows imaging larger volume with a temporal resolution of a few Hz. This enhancement in temporal resolution remains valid for high speed rotating mirrors (polygons, resonant scanners, etc.) and thus could potentially yield scan speeds on the order of a few kHz (Rajadhyaksha et al., 1999).

On the other hand, the cell loading method must also be optimized to eliminate contaminating background fluorescence. Hence, the expression of genetically-encoded proteins of interest in mice and targeted viral infection are methods of choice to be used with the extended DOF.

#### Application: time-lapse imaging of calcium fluctuations in thick samples

To demonstrate that this technique is compatible with live tissue imaging, we acquired a time-lapse sequence of calcium fluctuations in a thick, acute slice of mouse cortex stained with Fluo-4.

The biological sample used in this section is a thick acute slice of adult mouse cortex, stained with a calcium indicator. The sample preparation is detailed above. Fluorescence images of the calcium indicator Fluo-4 AM were taken at a frequency of 0.5 Hz (one frame every 2 s; 2 ms/line; 512 × 512 pixels) with the extended DOF set-up is described above. The excitation and emission light were separated by a dichroic mirror at 665 nm (Semrock). The emission light was truncated by a 633 nm short-pass filter (Semrock). For measurements of calcium-dependent changes, a sequence of fluorescence images was acquired. To stimulate cellular activity, the extracellular solution was switched from the standard solution to the high potassium solution every 60 s.

**Figure 7:**
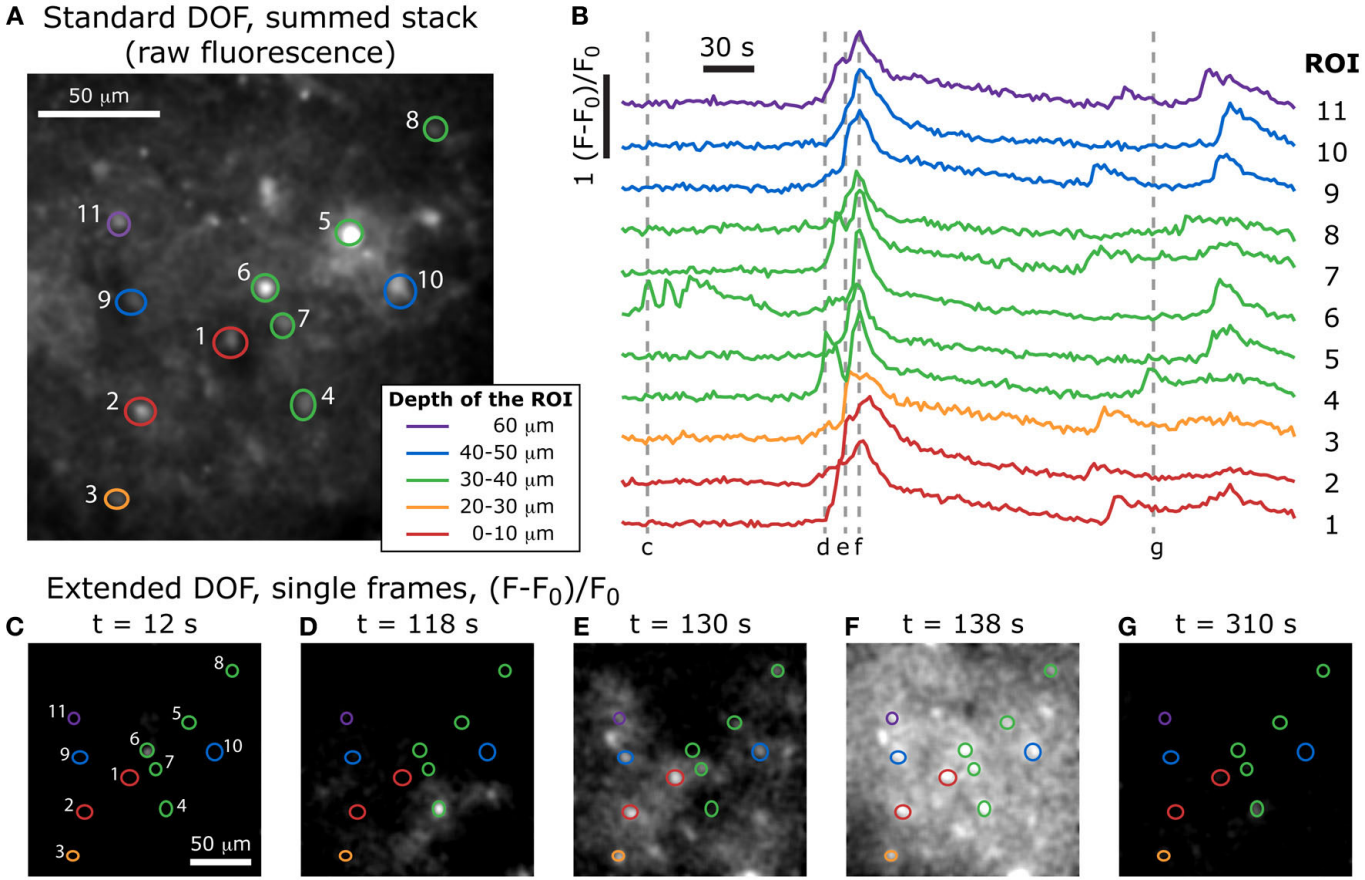
**Volumetric imaging of calcium dynamics in mouse cortex**. **(A)** Positions of the ROIs in the specimen, acquired with a standard two-photon stack at the end of the experiment (the summed stack of the raw fluorescence images is shown here), and color-coded to indicate the depth of each feature. **(B)** Single-cell calcium transients in an acute slice of mouse cortex stained with Fluo-4 AM, imaged with the extended DOF set-up and corresponding to the ROIs in **(A)**. Dotted lines correspond to the extended DOF single frames shown below. **(C–G)** (F-F_0_)/F_0_ single frames from the time-lapse acquisition of calcium dynamics (full video available online: Supplementary Movie S1).

In each sampling epoch, an average of the first 20 images was calculated to set the baseline value, F_0_. Regions of interest (ROI) were defined in the first image, and the normalized fluorescence changes (F-F_0_)/F_0_ were measured throughout the image sequence. For the time-lapse video of the data presented in Figure 7 (and Supplementary Video S1, available online), each frame was smoothed with a gaussian filter with a 2-pixel radius to reduce noise, brightness and contrast levels were also adjusted.

After the extended DOF time-lapse acquisition was completed, we reverted to the standard two-photon set-up by removing the axicon and lens and proceeded to acquire a stack of images with enough laser power to resolve the recorded cells. With this stack of images, it was possible to compare the features in each plane to the ones observed during the extended DOF time-lapse acquisition. We were therefore able to determine at which depth was located the bulk of the features selected in each ROI used for the extended DOF acquisition sequence. These depths are color-coded in Figure 7 (top) and the ROIs are superimposed with the summed stack from the standard DOF set-up.

We see in Figure 7 that several features located at different depths in the sample have calcium levels that are partially synchronized. For example, ROIs 1, 3, 7, 9, and 11 all show three main peaks of calcium concentration at approximately the same times, even though they span 60 μm in depth. We can therefore infer that the cells in these five regions are part of a common 3-dimensional network that receives synchronized inputs. It would have been very difficult to observe this with a standard DOF since, as discussed above, it would take at least 7 s to image a complete 60 μm-thick volume.

#### Comparison of high-speed microscopy approaches

Let us now compare our method's speed to that of three of the highest speed two-photon microscopy systems recently published.

The first one (Cheng et al., 2011) uses a resonant mirror for the fast-scanning axis to obtain frame rates of 250 Hz for single images with 500 × 500 pixels. They split the beam into *N* = 4 separate delay lines to multiplex the excitation temporally and add optical elements to these lines to separate the 4 beams axially, causing the excitation beam to be focused at 4 different depths during one pulse cycle. They therefore achieve a volume-scan rate of 250 Hz/N = 62.5 Hz, but a large portion of the volume is still not imaged since the DOF is approximately 0.8 μm and we wished to sample 60 μm depth. If this system were adapted to multiplex the excitation beam at *N* = 20 different depths, spanning 60 μm, it would then be equivalent to our experiment with the Bessel extended DOF microscope (although the complexity of aligning a set-up with 20 delay lines is highly challenging). The effective volume-scan rate of such a system would then be 250 Hz/N = 12.5 Hz, which is still three times faster than the fastest volume-scan rate of our set-up. Nevertheless, both the temporally multiplexed beam approach and the use of a resonant scanning mirror are fully compatible with our proposed approach and could be applied to further increase the volume scanning speed.

The second method with which we compare our system's performance is the random-access scanning two-photon microscope (Katona et al., 2012). This approach uses acousto-optic deflectors to steer the laser beam instead of oscillating mirrors. In the 3D line-scanning mode, this method can sample up to 500 points per kHz. A volume containing 500 × 500 × 20 voxels would then require an acquisition time of 10 s. In this case, our system is at least 20 times faster. Although it is slow for complete volume scans, the advantage of the random-access microscope is that once the user knows where the regions of interest lie, only a small subset of points must be sampled repetitively during the remainder of the experiment. On the other hand, the problem of focus drift or sample movement remains, so the random-access approach might not be appropriate for all experiments, whereas the Bessel-beam approach is more flexible.

The third fast two-photon method that we mention here uses a spatial light modulator to shape the two-photon illumination pattern in the focal plane (for example, see Nikolenko et al., 2008). SLM microscopy is ongoing and its speed is theoretically limited by the refreshing rate of the detecting module. The advantages and disadvantages of this method are similar to those of the random-access approach since it offers a very high throughput of information on a fixed number of cells, and a previous knowledge of the sample must be acquired and focus drift or sample movement could affect the recorded signal if they are not monitored and compensated in real-time.

Finally, let us remark on the choice of DOF. Although it could appear interesting to increase the DOF extensively in order to obtain a greater gain in speed, one should be aware that as the DOF increases, the probability of two or more labeled features being super-imposed also increases. The DOF should therefore be adjusted to the labeling density. Larger depths of field should only be used with sparsely labeled samples to avoid measurement errors from super-imposed cells.

### Simpler stereoscopic imaging

In the previous section, we mentioned that the information about depth cannot be retrieved from a single extended DOF image. A simple way to circumvent this disadvantage is to compose a stereoscopic pair by inducing a tilt in the focal line at the sample (Botcherby et al., 2006). With an extended DOF set-up, it is possible to acquire a stereoscopic image with only two x-y scans, one for each viewpoint. To illustrate this, we present an example of stereoscopic imaging with a sample of protein-labeled neurons from a fixed slice of mouse cortex.

**Figure 8:**
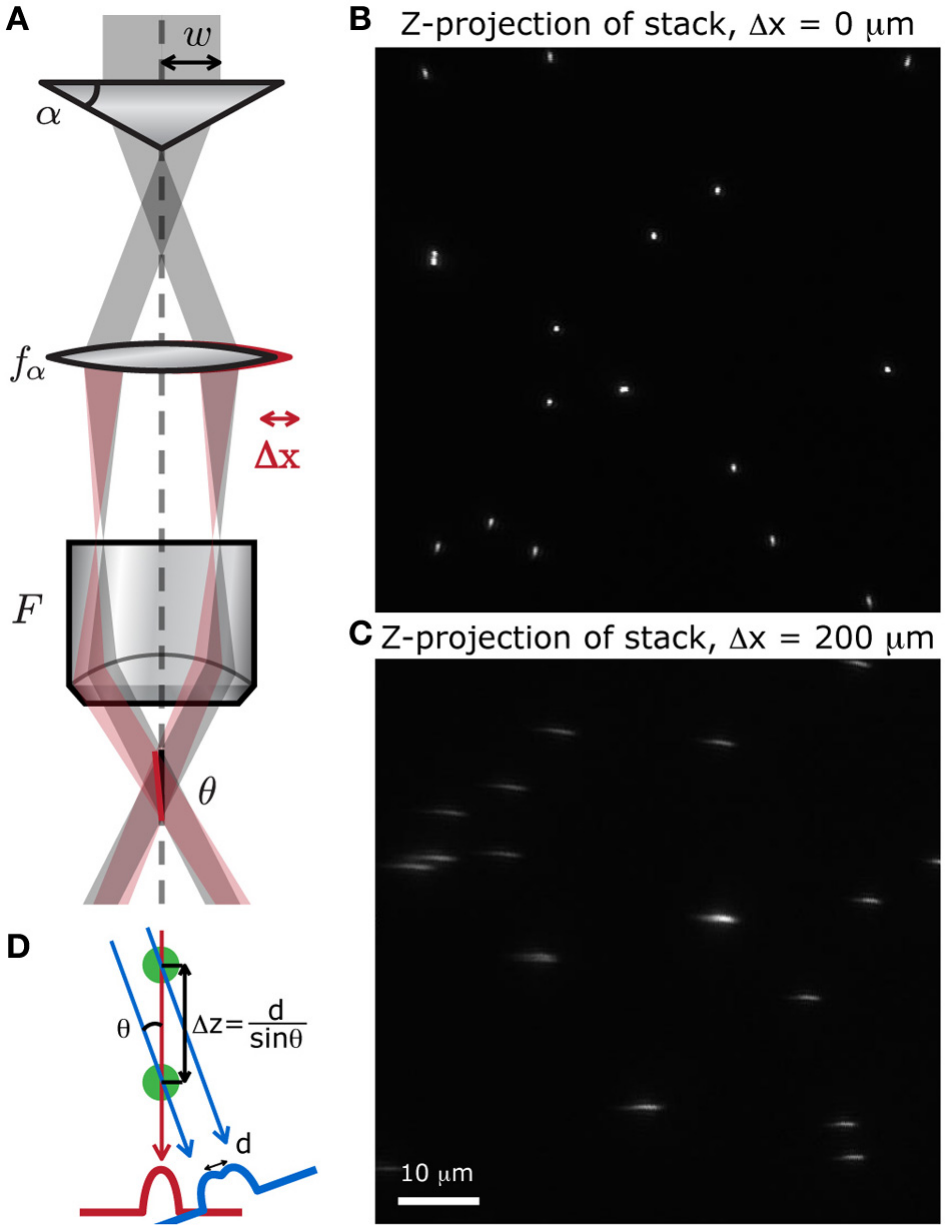
**Simple method to produce a stereoscopic pair of images**. **(A)** A small displacement of either the lens after the axicon induces a tilt θ of the focal line at the sample, with respect to the optical axis. We scanned a random distribution of small fluorescent beads (*Molecular* *Probes*, Fluosphere 505/515, diameter 500 nm) at different depths, spanning 60 μm, to produce a stack of images. The z-projection of these stack show that when **(B)** no shift is applied, the point of view is perfectly vertical, and when **(C)** the lens is slightly shifted, the point of view is tilted with respect to the vertical. **(D)** Two superimposed cells can be distinguished with this method if they are separated by a distance Δz = d/sinθ.

The approach we used to induce a tilt in the focal line is illustrated in Figure 8A. When a lateral shift Δ*x* is applied to either the axicon or its associated lens, the ring of light incident on the back aperture of the objective is shifted, and the focal line is tilted with respect to the optical axis with the parallax angle θ, defined below (adapted from Botcherby et al., 2006):

(2) $$\theta =\left(\frac{f_{2}}{f_{1}F}\right)\frac{\Delta x}{\cos\left(m\beta \right)}$$

To verify that displacing the lens does induce a tilt in the focal line throughout the field of view, we acquired a stack of images from fluorescent microspheres (Molecular Probes, Fluosphere 505/515, diameter 500 nm) mounted on a 150 μm thick coverslip with fluorescent mounting medium (Dako). A z-projection of this stack shows whether or not the point of view is shifted. When there is no shift, the point of view is perfectly vertical and all the beads appear as regular circles (Figure 8B). When the lens is displaced, the focal line is shifted and the beads appear stretched out along the tilted axis (Figure 8C). We can also see from this projected image that the transverse resolution of the system has not been degraded by the displacement of the lens.

The biological sample used in this section is a fixed slice of cortex from a transgenic mouse in which genetically encoded fluorescent markers are expressed in a subset of cells. The sample preparation is detailed in Section Sample Preparation.

**Figure 9:**
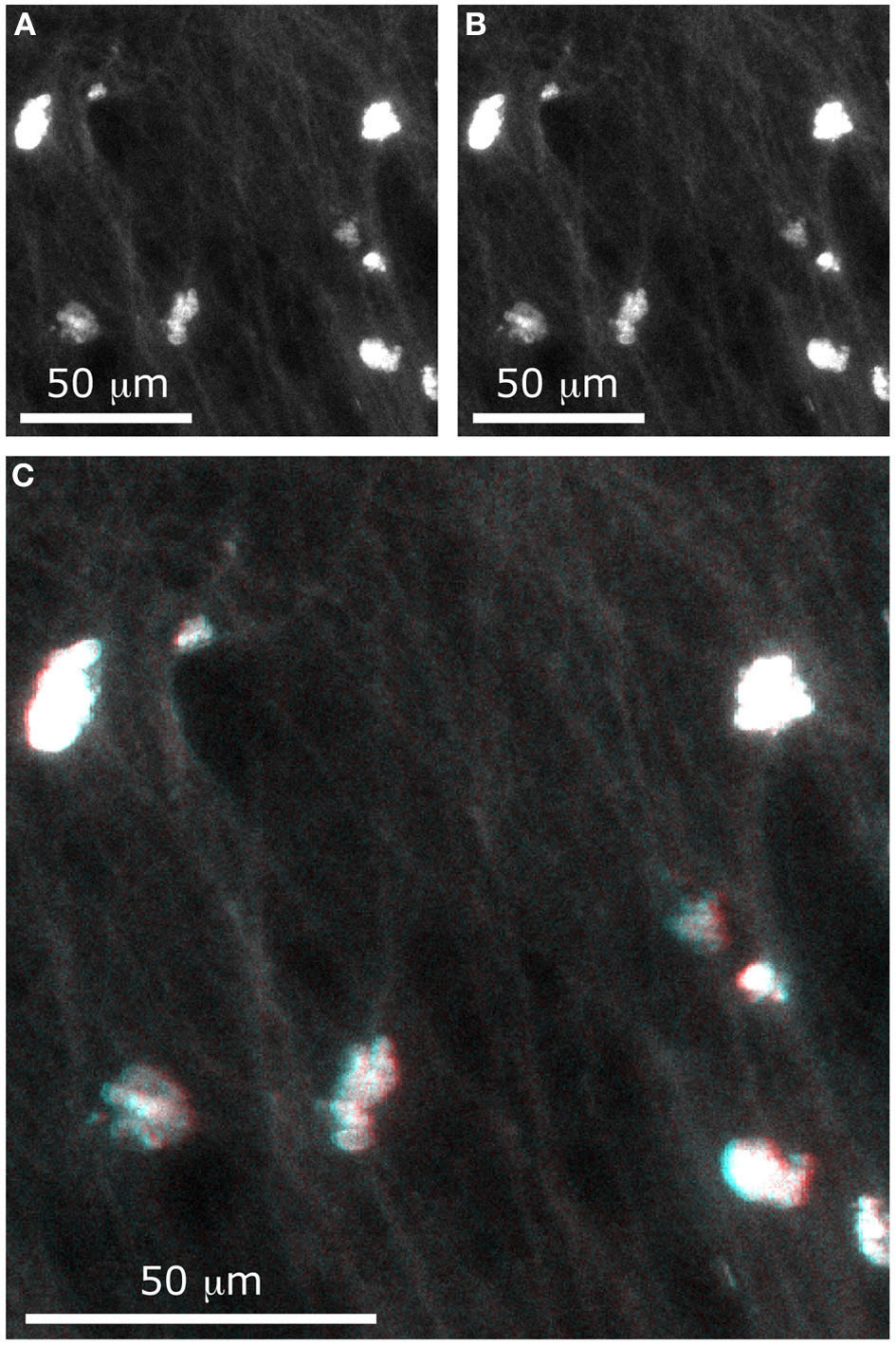
**Stereographic imaging of neurons in only two frames**. With the extended DOF microscope, **two** images were acquired, with different values of Δx: **(A)** Δx = −100 μm and **(B)** Δx = −100 μm. The composite image **(C)** can be viewed with 3-D perception using red-cyan glasses.

A set of two images was acquired with the extended DOF system in order to get a stereoscopic image. To displace the lens, we mounted it on a computer-controlled motorized translation stage. For the first image (Figure 9A), the lens was displaced by Δ*x* = −100 μm, inducing a 2.5° tilt in the focal line. For the second image (Figure 9B), the lens was displaced by Δ*x* = 100 μm, inducing a 2.5°tilt in the focal line. To improve the signal-to-noise ratio, each line was averaged 10 times in both images.

Although a stereoscopic pair would better be viewed using a 3D display and the matching goggles, we have chosen to present our results as a red-cyan anaglyph. To form a stereogram that can be viewed with red-cyan glasses, a black-to-red colormap was assigned to the first image and a black-to-cyan colormap was assigned to the second image. Adding the two images formed the composite image shown in Figure 9C. When viewing this image with the appropriate glasses, one can see that the cell bodies are located at different depths (for example, the cell at the lower right of the image appears much higher than the one at the upper left). Even small details such as the fine dendrites are resolved with depth perception.

With this method, it is possible to recover information about relative depths. As illustrated in Figure 8D, when two cells are superimposed in the z-axis, it is possible to distinguish them if they are separated by a distance Δz = d/sinθ, where d is the cell diameter. For example, when the total tilt between the two images is θ = 10° and if we approximate a cell body to a 10-micron sphere, then the minimal distance at which two superimposed cells can be distinguished is Δz = 60 μm. With these parameters, the cell bodies would appear as a single circle in one image and as two touching circles in the second image.

To produce the same image with a standard two-photon microscope, it is necessary to acquire a stack of at least 30 images (one image every 2 μm, spanning 60 μm) and to recompose the left and right image by calculating projections of the stack according to two different angles. All of these steps and calculations are necessary to generate a similar composite image comparable to the one obtained in Figure 9C with only two scanned frames. The method we presented here, using an axicon, is therefore dramatically simpler and faster, and could be implemented into a conventional two-photon microscopy system with a 3D screen to view samples stereoscopically in real time.

## Discussion

In this paper, we have presented a simple modification to the standard two-photon microscope, which consists in extending the DOF of the system without compromising on transverse resolution, by adding two optical elements in the laser beam path: an axicon (conical lens) and a regular lens.

With this modification, we performed measurements on three different types of biological samples, all of which are commonly used in the field of neurosciences: a thin sample of cells grown in culture on a glass coverslip; a thick, live sample of acute brain slice from a mouse; a thick, fixed sample of transgenic mouse cortex. The first sample was labeled by transfection of a green fluorescent protein, the second sample was stained with a calcium-ion indicator to track cell activity *ex vivo* with variations in fluorescence intensity, and the third sample contained genetically encoded fluorescent markers expressed in a subset of cells. All of these marking techniques are common tools in neuroscience, which shows that the extended DOF system is compatible with the current biological techniques.

With each sample, we highlighted the benefits of using an extended DOF system based on a Bessel beam, when compared to the standard two-photon microscope. For thin samples, or specimens in which most features of interest are generally located in the same plane, we have shown that the extended DOF provides a more stable focus, which can protect against vibrations or focus drift. The same benefit could be exploited for *in vivo* measurements, to avoid measurement biases due to small movements (e.g., due to breathing). For thick samples where the features of interest are dispersed into a 3-dimensional matrix, an extended DOF improves the speed of volumetric scans (up to 30 times faster), which allows resolving dynamics on a shorter time-scale. Despite the fact that the information about depth is lost, we showed that it is possible to recover this information at the end of the experiment by removing the DOF extension add-on, reducing the DOF of the set-up to a standard two-photon microscope. Finally, we presented stereoscopic imaging in fixed tissue, a far simpler and faster way of obtaining depth information than with the standard image stack method. We introduced an efficient approach to achieve the stereoscopic image with minimal degradation of the focal line, in which only two images needed to be acquired, instead of a complete stack (e.g., 60 images) and a 3-D reconstruction algorithm required with a standard two-photon system.

Further extension of the DOF could be envisaged, however this comes with power limitations as the power is distributed along the focal line. Furthermore, given that this is a two-photon effect, the power loss is squared with the increase in focal line. The maximum focal length extension possible is thus limited by the power of the laser source available, by the density of the labeled features and by the fluorescence collection capabilities of the system (Sergeeva et al., 2010). Yet, phototoxicity or photobleaching are not necessarily increased if the power is tuned so that the fluorescence signal remains the same at each point along the focal line. The same peak excitation intensities at each point within the sample can thus be achieved with both the Bessel beam and a conventional Gaussian beam.

A critical advantage of the proposed approach is that it allows integration of the axicon into a standard laser-scanning microscope. The system is thus fully retrofittable into existing commercial systems and can be designed to offer both Bessel beam and Gaussian beam illumination in the same system, allowing both types of imaging to be performed sequentially on the same sample to exploit the advantage of both techniques at the same time. This key feature will likely result in a broad acceptance of the technology by the community, further amplifying and accelerating its impact. We believe that due to the flexibility, simplicity and accessibility of the extended DOF method, combined with all the benefits it provides (steadier focus, faster volume scans and simpler stereography), this technology will have a transforming impact for life sciences in general.

### Conflict of interest statement

The authors declare that the research was conducted in the absence of any commercial or financial relationships that could be construed as a potential conflict of interest.

## Supplementary material

Movie S1 **Extended DOF two-photon imaging of calcium transients in an acute slice of adult mouse cortex**. (F-F_0_)/F_0_ images from the x-y-t time-lapse sequence of a 190 × 190 μm scan field depict single-cell calcium transients in an acute slice of mouse cortex stained with Fluo-4 AM, imaged with the extended DOF set-up.
